# A nationwide survey of the opinions of paediatricians regarding the management of neonates born to women with group B *Streptococcus* in Japanese maternity homes

**DOI:** 10.1186/s13104-019-4526-3

**Published:** 2019-08-01

**Authors:** Kotomi Yamaguchi, Kazutomo Ohashi

**Affiliations:** 1 School of Medicine Nursing Course, Gifu University, 1-1 Yanagido, Gifu, 505-1194 Japan; 2 Department of Children and Women’s Health, Division of Health Sciences, Osaka University Graduate School of Medicine, 1-7 Yamadaoka, Suita, 565-0871 Japan

**Keywords:** Paediatricians, Group B *Streptococcus*, Maternity home, Surveillance, Japan

## Abstract

**Objective:**

The Japanese Midwifery Association (JMA) guidelines allow midwives to manage group B *Streptococcus* (GBS)-positive women during pregnancy and labour at maternity homes. However, no guidelines exist to manage neonates born to GBS-positive women in Japan. We aimed to investigate the opinions of paediatricians regarding optimal management strategies for neonates born to GBS-positive women in maternity homes. A questionnaire was sent to paediatricians at 396 Japanese perinatal medical centres. We examined opinions regarding examinations and routine clinical tests for neonates born to GBS-positive women in maternity homes.

**Results:**

Of 235 paediatricians, only 11.2% considered that paediatric examinations were unnecessary for neonates born to GBS-positive women in maternity homes. Moreover, 20.5%, 13.2%, and 11.1% of paediatricians considered culture test of the nasal cavity, serum C-reactive protein level analysis, and blood cell count analysis, respectively, necessary for neonates born to GBS-positive pregnant women with intrapartum antibiotic prophylaxis (IAP), whereas 36.3%, 56.2%, and 40.6% of paediatricians considered these tests necessary in cases without IAP. The JMA guidelines had low penetration rates among paediatricians in Japan. To manage neonates born to GBS-positive women in maternity homes, midwives should engage with commissioned paediatricians in more detail and develop appropriate strategies to increase awareness and cooperation.

## Introduction

Group B *Streptococcus* (GBS) causes sepsis, meningitis, and pneumonia in neonates. Early-onset GBS (EOGBS) is defined as the condition that occurs within 6 days of birth. In their 1996 guidelines, the Centers for Disease Control and Prevention (CDC) recommended primary prevention with universal screening for maternal GBS colonisation and the use of intrapartum antibiotic prophylaxis (IAP), and in 2010, it recommended the secondary prevention of EOGBS [1]. Following a study involving universal antenatal GBS screening for pregnant women, the Japan Society of Obstetrics and Gynecology (JSOG) also issued recommendations for IAP [2].

Midwives in Japan cannot legally provide prescriptions or conduct any clinical test by themselves. In fact, the 2009 guidelines published by the Japanese Midwives Association (JMA) stated that midwives can not handle GBS-positive women during pregnancy and labour in maternity homes until 2013. In 2014, the JMA revised their guidelines to allow midwives in maternity homes to manage GBS-positive women during pregnancy and labour only if they complied with the JSOG guidelines and directions of commissioned obstetricians and paediatricians working for cooperative medical facilities [3]. A 2016 study reported that approximately 6000 neonates were born in maternity homes that were not attended by obstetricians or paediatricians; this represented 0.6% of the total deliveries that year [4].

The CDC guidelines describe the management of cases with signs of neonatal sepsis for preventing EOGBS [5]. When signs of sepsis are observed, neonates undergo clinical tests and antibiotic therapy. As there is no medical doctor at maternity homes in Japan, there is a possibility of a delay in examination, testing, treatment, and transportation when septic signs appear. In addition, there are no guidelines for managing neonates born to GBS-positive women in Japan.

To consider the management of neonates born to GBS-positive women in Japanese maternity homes, we examined the opinions of paediatricians regarding the JMA guidelines, the timing of neonatal examination, and routine clinical tests for neonates born to GBS-positive women. This represents the first survey of paediatricians’ opinions regarding the guidelines for the prevention and management of GBS-positive women in pregnancy and labour and of the neonates they deliver in Japanese maternity homes.

## Main text

### Methods

Between October and November 2016, we conducted an anonymous survey of all 396 perinatal medical centres shown in the 2015 register held by the Ministry of Health, Labour and Welfare, Japan. We sent a self-administered questionnaire and explanatory document to each centre by mail. The questionnaires were answered by a representative paediatrician from each centre.

The questionnaire included several characteristics of the responders (including their years in practice as paediatricians, experience in treating neonatal GBS disease, and experience in treating neonates transferred from maternity homes), background of the facility (annual data for livebirths and neonatal transfers), opinions on universal screening and secondary prevention, recognition of the existence of the JMA guidelines and its revision in 2014, handling of deliveries in maternity homes, and timing of paediatric examinations for neonates born in maternity homes. We also created an original questionnaire for routine clinical tests that paediatricians consider necessary for neonates born to GBS-positive women with or without the administration of IAP, with reference to previous studies [6, 7] and JSOG and CDC guidelines. These guidelines are in Japanese.

The Ethical Committee of Kyoto University Medical School approved this study (Approval number: R0840). The return of a questionnaire from a paediatrician was considered as the provision of consent. The anonymity of the participants was preserved.

#### JSOG guidelines for managing pregnant women with GBS infection

1. Conduct the GBS screening test between 33 and 37 weeks of gestation (this guideline was revised to “between 35 and 37 weeks of gestation” in 2017).
2. Collect a specimen for GBS culture via a lower vaginal and anal canal swab.
3. Administer prophylactic antibiotic treatment to GBS-positive women who are in labour and scheduled to give birth via vaginal delivery or after premature rupture of membranes during pregnancy.

#### Recommendations for managing GBS-positive women in pregnancy or labour (issued by the JMA in 2014)

Even when medical doctors are absent, midwives alone can manage GBS-positive women in labour at maternity homes only if they comply with the JSOG guidelines and cooperate with commissioned obstetricians and paediatricians working for the commissioned facilities to respond to unforeseeable events.

#### Statistical analysis

For each question, we created a frequency distribution for statistical analysis. Descriptive statistical analyses were carried out by using IBM SPSS Statistical Software version 23.0. (IBM Japan Ltd., Tokyo, Japan).

### Results

We sent questionnaires to 396 perinatal medical centres by mail and received responses from 236 (59.6%) centres, including one facility that was not designated as a perinatal medical centre at the time of the study. Finally, we included 235 responses (a valid response rate of 59.3%) in our final analyses.

The mean duration (years) that the respondents had experienced in practice as paediatricians was 20.6 ± 7.6 years (N = 232). Of these, 94.9% and 74.9% had experience in treating neonates with GBS and receiving neonates transferred from maternity homes because of several abnormal conditions, respectively. The mean annual number of livebirths and neonatal transfers was 600.0 ± 341.7 (N = 227) and 89.3 ± 129.3 (N = 195), respectively.

Most paediatricians (97.5%, 230/235) agreed with universal screening for GBS in pregnant women according to the JSOG guidelines. In addition, 77.0% (181/235) considered that guidelines for neonates born to GBS-positive women were needed.

Sixty-two paediatricians (26.4%) recognised the existence of the JMA guidelines but only 23 (9.8%) knew that midwives could manage GBS-positive women in labour at maternity homes, as described by the 2014 revision of the JMA guidelines. Regarding deliveries in maternity homes, 46.0% of respondents stated that were some general problems whereas 50.2% stated that there was no problem.

**Fig. 1:**
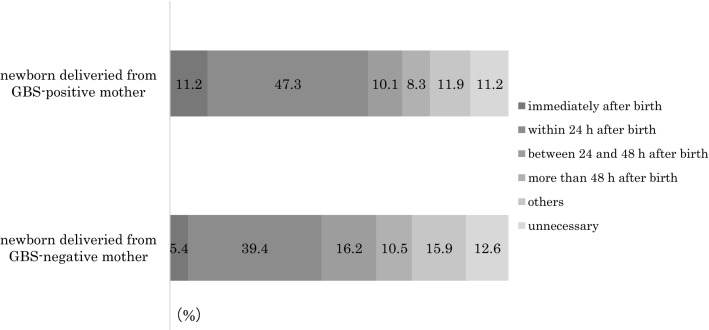
Opinions of paediatricians regarding the timing of paediatric examinations for neonates in maternity homes (N = 235)

Only 11.2% and 12.6% of respondents considered that paediatric examinations were not necessary for neonates born to GBS-positive and GBS-negative women in maternity homes, respectively. Furthermore, 68.6% and 61.0% of respondents considered that paediatric examinations should be conducted within 48 h of birth in cases of GBS-positive and GBS-negative women, respectively (Fig. 1).

**Fig. 2:**
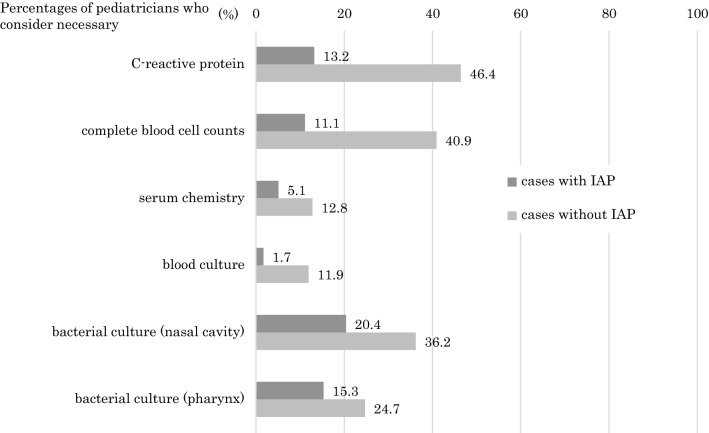
Proportions of paediatricians who consider routine clinical tests necessary for neonates born to GBS-positive mothers (N = 235, multiple answers)

We examined routine clinical tests that paediatricians considered necessary for neonates born to GBS-positive women in cases with or without the administration of IAP. The CDC guidelines refer to ‘appropriate’ or ‘inappropriate’ cases, and cases in which IAP is implemented > 4 h before delivery are considered ‘appropriate’. In contrast, the JSOG guidelines do not refer to the timing of IAP administration; thus, we used the terms ‘with’ or ‘without’. In cases administered with IAP, 20.5%, 13.2%, and 11.1% of respondents considered a culture test of the nasal cavity, serum C-reactive protein (CRP) level analysis, and complete blood count (CBC) analysis, respectively, to be necessary. In contrast, 36.3%, 56.2%, and 40.6% of respondents considered these tests to be unnecessary in cases without IAP, respectively (Fig. 2).

### Discussion

Recent studies have reported that the use of IAP for GBS-positive women in labour effectively reduced the incidence of EOGBS [8, 9]. Following publication of the JSOG guidelines, the incidence of EOGBS has not changed in Japan although the mortality rate associated with this condition has decreased [10]. Therefore, most of our survey respondents (97.5%) agreed with universal antenatal screening, whereas 77.0% expected novel evidence-based guidelines for neonates born to GBS-positive women. However, only 9.8% of our respondents were aware of the JMA guidelines and the 2014 revision, indicating that most paediatricians were unaware that midwives working in maternity homes could manage GBS-positive women during pregnancy and labour. We previously reported that 66.2% of maternity homes handled women with GBS in pregnancy and labour [11]. However, there are no guidelines for the management of neonates born to GBS-positive women in maternity homes. The CDC guidelines state that it is necessary to observe apparently healthy neonates for > 48 h if their mothers had received inadequate IAP. In our present study, 68.6% and 61.0% of respondents considered paediatric examinations within 48 h to be necessary for neonates born to GBS-positive and GBS-negative women. Only 11.2% and 12.6% of respondents considered paediatric examinations to be unnecessary. In addition, 46.0% of respondents stated that there were some general problems encountered during deliveries in maternity homes. To manage GBS-positive women during labour in maternity homes, midwives should inform paediatricians working for the commissioned facilities regarding the guidelines and take time to discuss the management of neonates born to GBS-positive women.

According to our previous study, IAP was implemented for GBS-positive women in 82.2% of maternity homes in accordance with the JMA guidelines [12]. In the present study, > 79.6% of our respondents considered each laboratory test unnecessary for neonates born to GBS-positive mothers with IAP, whereas > 46.4% and 40.9% considered serum CRP level and CBC tests, respectively, necessary for mothers without IAP. Measuring serum CRP level has previously been reported to be a useful predictor of EOGBS in Japan [13]. However, 46.2% of our respondents considered it necessary to investigate serum CRP level in mothers without IAP. A previous study reported that CBC test and blood cultures were not useful for diagnosing EOGBS in cases where IAP had been inadequate [13]. In the present study, 40.6% of respondents considered CBC test to be necessary in cases without IAP. A previous meta-analysis of cases without IAP estimated that the GBS vertical transmission rate was 36.4% and that EOGBS occurred in 3.0% of cases [14]. Therefore, detecting GBS may have been considered important to prevent vertical transmission and EOGBS. Indeed, 20.5% and 15.4% of our current respondents considered cultures of specimens from the nasal cavity and pharynx to be necessary. Unfortunately, bacterial culture tests after birth are associated with poor positive productivity and high costs and yield little information regarding to potential bacterial sepsis [15]. These results suggest that standard routine clinical tests should be immediately considered in cases of neonates born to GBS-positive women in Japan. There is a clear need for further discussion regarding to the management of neonates after birth with the commissioned paediatricians when handling the deliveries of GBS-positive women in maternity homes in Japan.

### Conclusion

Although most respondent paediatricians agreed with IAP following universal antenatal screening for GBS, they did not recognize the JMA guidelines. Therefore, midwives in maternity homes should make more efforts to promote the recognition of the JMA guidelines among paediatricians. In addition, as there are no guidelines related to the secondary prevention of GBS vertical infection in Japan, it is now necessary for midwives in maternity homes to discuss potential management strategies with the commissioned paediatricians, including paediatric examinations and clinical tests for neonates born to GBS-positive women. There is a clear need for more publicity of the JMA guidelines and its latest revision, and greater levels of cooperation between midwives and commissioned obstetricians and paediatricians.

## Limitations

There are some limitations to this study that should be considered. First, we examined one paediatrician at each perinatal medical centre, and his/her opinion might not be representative of all staff. Second, the response rate was only moderate (59.3%). However, 94.9% of the participating paediatricians had treated neonates for GBS, and 74.9% had managed neonates transferred from maternity homes. Therefore, we consider that our results are both robust and valuable.

## Data Availability

The datasets used and/or analysed during the current study are available from the corresponding author on reasonable request.

## Abbreviations

CDC: Centers for Disease Control and Prevention
CRP: C-reactive protein
EOGBS: early-onset GBS disease
GBS: group B *Streptococcus*
IAP: intrapartum antibiotic prophylaxis
JSOG: Japan Society of Obstetrics and Gynecology
JMA: Japanese Midwives Association
PROM: premature rupture of membranes
